# Liposome Delivery of Nucleic Acids in Bacteria: Toward *In Vivo* Labeling of Human Microbiota

**DOI:** 10.1021/acsinfecdis.1c00601

**Published:** 2022-06-23

**Authors:** Luís Moreira, Nuno M. Guimarães, Sara Pereira, Rita S. Santos, Joana A. Loureiro, Maria C. Pereira, Nuno F. Azevedo

**Affiliations:** † LEPABE - Laboratory for Process Engineering, Environment, Biotechnology and Energy, Faculty of Engineering, University of Porto, Rua Dr. Roberto Frias, 4200-465 Porto, Portugal; ‡ ALiCE - Associate Laboratory in Chemical Engineering, Faculty of Engineering, University of Porto, Rua Dr. Roberto Frias, 4200-465 Porto, Portugal

**Keywords:** microbiome, fluorescence *in vivo* hybridization, nucleic acid probes, liposomes

## Abstract

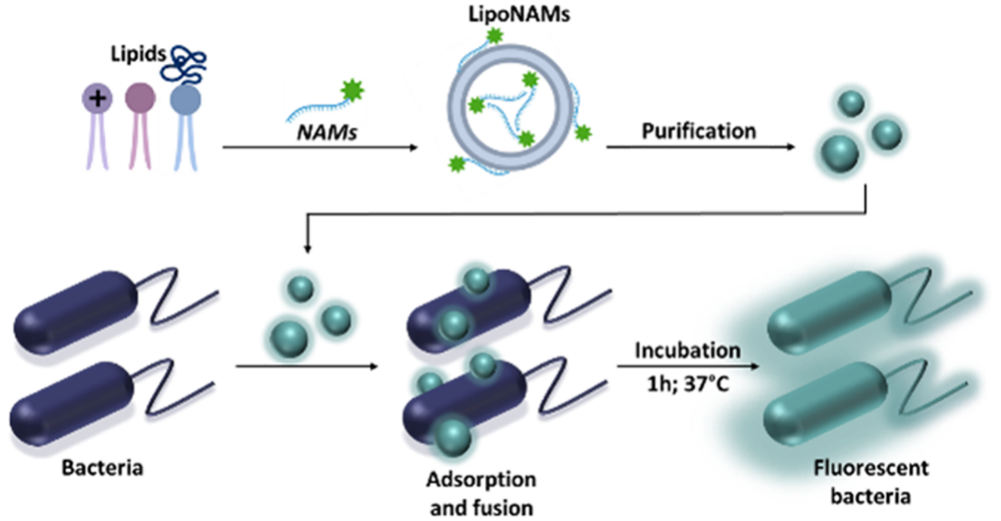

Development of specific probes to study the *in vivo* spatial distribution of microorganisms is essential to understand the ecology of human microbiota. Herein, we assess the possibility of using liposomes loaded with fluorescently labeled nucleic acid mimics (LipoNAMs) to image Gram-negative and Gram-positive bacteria. We proved that liposome fusion efficiencies were similar in both Gram-negative and Gram-positive bacteria but that the efficiency was highly dependent on the lipid concentration. Notably, LipoNAMs were significantly more effective for the internalization of oligonucleotides in bacteria than the fixation/permeabilization methods commonly used *in vitro*. Furthermore, a structural and morphological assessment of the changes on bacteria allowed us to observe that liposomes increased the permeability of the cell envelope especially in Gram-negative bacteria. Considering the delivery efficiency and permeabilization effect, lipid concentrations of approximately 5 mM should be selected to maximize the detection of bacteria without compromising the bacterial cellular structure.

The human body is a reservoir of complex microbial communities that establish host–microbe interactions with profound implications in health and disease.^1^ As our understanding of host–microbe ecology improves, it is expected that the microbiota will become a valuable resource for novel diagnostic and therapeutic approaches.^2,3^ As a consequence, the human microbiome has been extensively characterized in the last decades, providing detailed information regarding the diversity and abundance of microbial communities.^3−6^ Biopsy samples collected from patients have also provided snapshots of the spatial distribution of microorganisms before downstream characterization.^1,6,7^ However, studying gut microbiota in biopsy samples has several limitations such as the laxative preparation of the bowel that significantly changes microbiota, insufficient biomass, contaminations while handling the samples, and risk of bleeding and infection. This strategy is also unsuitable for the examination of microbiota in healthy volunteers due to the invasive character of the technique.^8^

As an alternative, *in vivo* labeling of microbiota could enable identification and spatial location of microorganisms in their native environment with minimal bowel preparation and risks to people. For this purpose, several optical probes are being designed to image bacteria in *in vivo* models. These probes consist of fluorescently labeled ligands (usually emitting in the infrared spectrum) that target bacterial surface structures.^9−12^ For instance, Akram et al.^13^ already demonstrated the feasibility of optical endomicroscopy to image Gram-negative bacteria in the lungs of human patients upon administration of fluorescently labeled polymyxin. However, none of the above-mentioned techniques are able to discriminate species or strains. This level of specificity is generally achieved by the identification of specific nucleic acid sequences inside the microbial cells. In previous works of our group, Fontenete et al.^14^ applied a fluorescently labeled locked nucleic acid (LNA) and 2′-*O*-methyl RNA (2′OMe) mixmer with phosphorothioate (PS) internucleoside linkages to detect *Helicobacter pylori* colonizing the mouse stomach, and Santos et al.^15^ demonstrated the ability of post-PEGylated DOTAP/DOPE liposomes to deliver LNA/2′OMe mixmers in *Helicobacter pylori* in the presence of native gastric mucus.

Here, we expand our earlier work and develop a new optical probe based on liposome-loaded nucleic acid mimics (LipoNAMs) to image bacteria belonging to the human microbiota genera *Escherichia*, *Pseudomonas*, *Listeria*, and *Staphylococcus*. In addition, we present a comprehensive study of the interaction of PEGylated DOTAP/DOPE liposomes with both Gram-negative and Gram-positive bacteria and evaluate the delivery efficiency of liposome-loaded LNA/2′OMe/PS oligonucleotides in these bacteria. The interaction of the liposomes with the bacterial envelopes was evaluated by studying the fusion efficiency and its dependence on lipid concentration. The ability of the liposomes to deliver the loaded nucleic acid mimics (NAMs) into the bacterial cytosol was then characterized, according to the concentration of lipids and NAMs in the liposomes. Finally, a structural and morphological evaluation of bacteria was carried out to clarify the delivery mechanism for both types of bacteria.

## Results

### Characterization of Unloaded Liposomes and LipoNAMs by Dynamic Light Scattering

**Figure 1:**
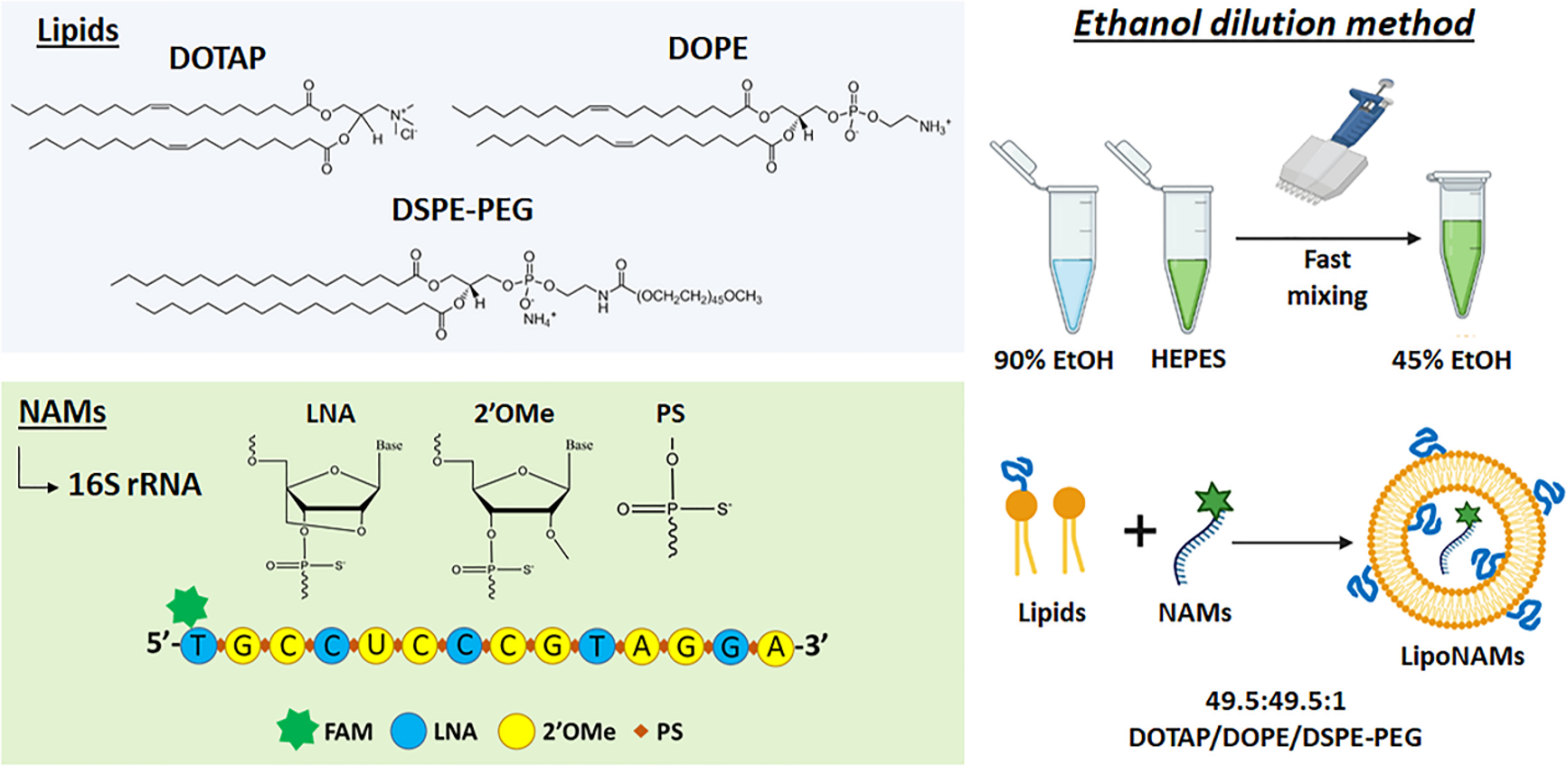
Structures of DOTAP, DOPE, and DSPE-PEG lipids. Sequence of the oligonucleotide probe (T-thymine, G-guanine, C-cytosine, U-uracil, and A- adenine) and structures of LNA, 2′OMe, and PS modifications (left). Schematic representation of the production of LipoNAMs using the ethanol dilution method (right).

Figure 1 presents the chemical structures of the lipids and NAMs, together with a schematic representation of the production of the LipoNAMs using the ethanol dilution method. Unloaded PEGylated liposomes (LS) were produced with a hydrodynamic diameter, PDI, and zeta-potential of 123 ± 14 nm, 0.20 ± 0.03, and 38 ± 4 mV, respectively. Loading different concentrations of NAMs (L10/N0.5 and L10/N1, where L10 corresponds to 10 mM lipids and N0.5 and N1.0 correspond to 0.5 and 1.0 μM FAM-labeled NAMs, respectively) had no significant impact on the size of the liposomes (Table S1). However, the increase in the concentration of NAMs reduced the mean surface charge to 2 and 5 mV for L10/N0.5 and L10/N1, respectively, which is likely due to the complexation of the negatively charged NAMs to the liposomes surface (Table S1). All formulations showed a PDI lower than 0.22, indicating that the ethanol dilution method is suitable to produce small and monodispersed cationic lipid vesicles. We then evaluated how the initial concentration of the NAMs in solution (5 and 10 μM) could influence the loading efficiency (%LE) (Table S1). For both concentrations the mean %LE ranged from 41 to 51%, which is in agreement with the literature.^16^

The hydrodynamic diameter, PDI, and zeta-potential of unloaded rhodamine-labeled liposomes (Rh-LS) varied between 111 and 117 nm, 0.17–0.20, and 36–40 mV, which are similar to those obtained for LS alone.

### Interaction between Rhodamine-Labeled Liposomes (Rh-LS) and Bacteria

**Figure 2:**
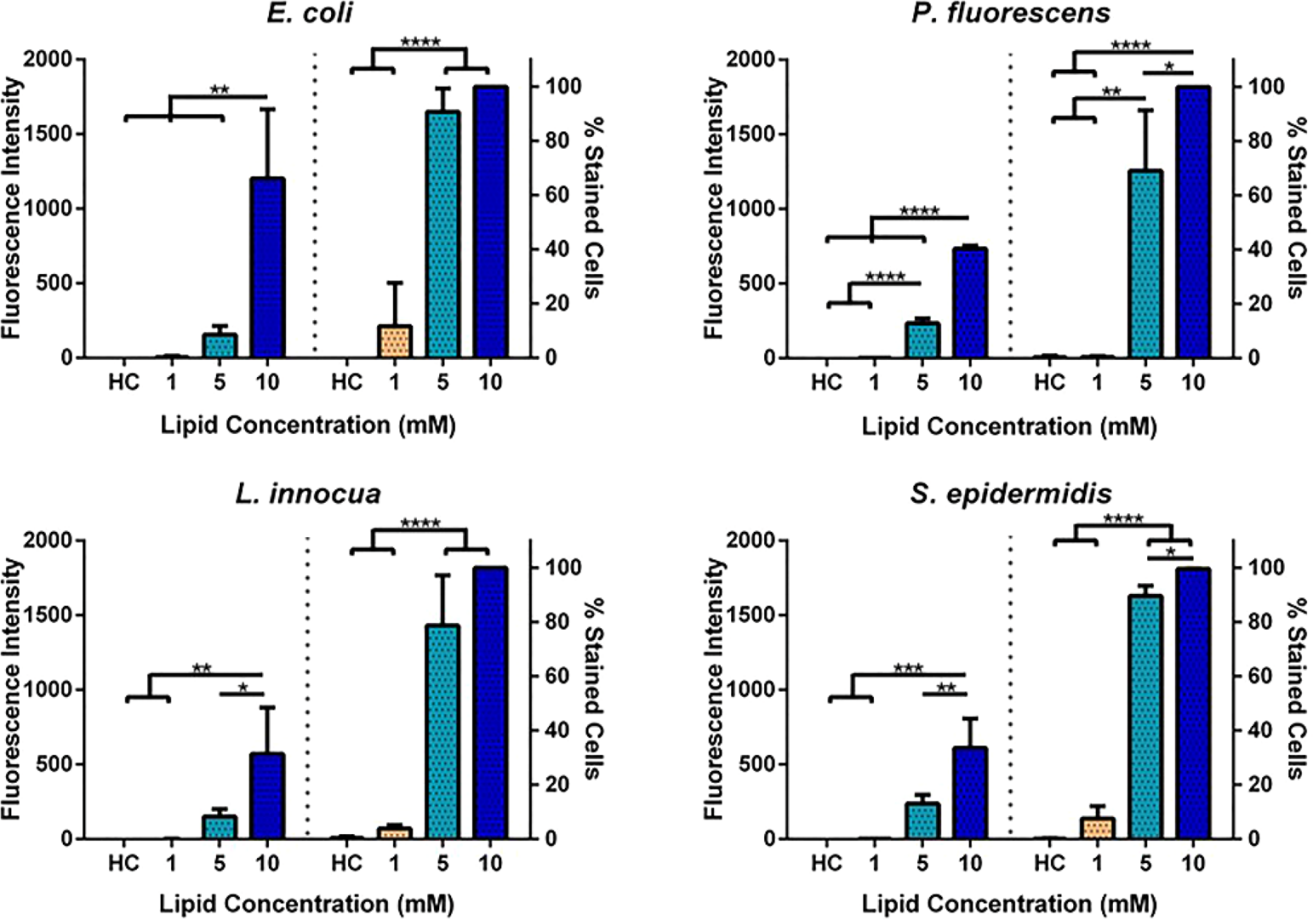
Quantitative analysis of the liposome–bacteria interaction in *E. coli*, *P. fluorescens*, *L. innocua*, and *S. epidermidis*. Unloaded rhodamine-labeled liposomes (Rh-LS) at 1, 5, and 10 mM (DOTAP/DOPE concentration) were used, each containing 50 μM Rh-PE lipid. Fluorescence intensity quantified by flow cytometry and normalized in relation to the control bacteria in HEPES (HC, HEPES control). Percentage of stained cells (% Stained Cells) as determined by cytometric analysis. The results are plotted as mean ± standard deviation of three repeated assays. Differences are statistically significant when *P* < 0.05 (*P < 0.05; **P < 0.01; ***P < 0.001; ****P < 0.0001). Brackets and lines indicate the groups being compared.

The interaction of cationic liposomes with Gram-negative and Gram-positive bacteria was assessed by flow cytometry upon exposure to Rh-LS followed by washing with a low concentration (0.1% v/v) of detergent solution to remove liposomes that were adsorbed (and not fused)^15^ to the bacterial envelope’s surface. The fusion efficiency was characterized by the quantification of the fluorescence intensity and the percentage of stained bacterial cells. To test if the ability of the liposomes to fuse with the bacterial envelope would be lipid-dose-dependent, bacteria were exposed to increasing lipid concentrations of the Rh-LS while the rhodamine concentration was kept constant (Figure 2).

**Figure 3:**
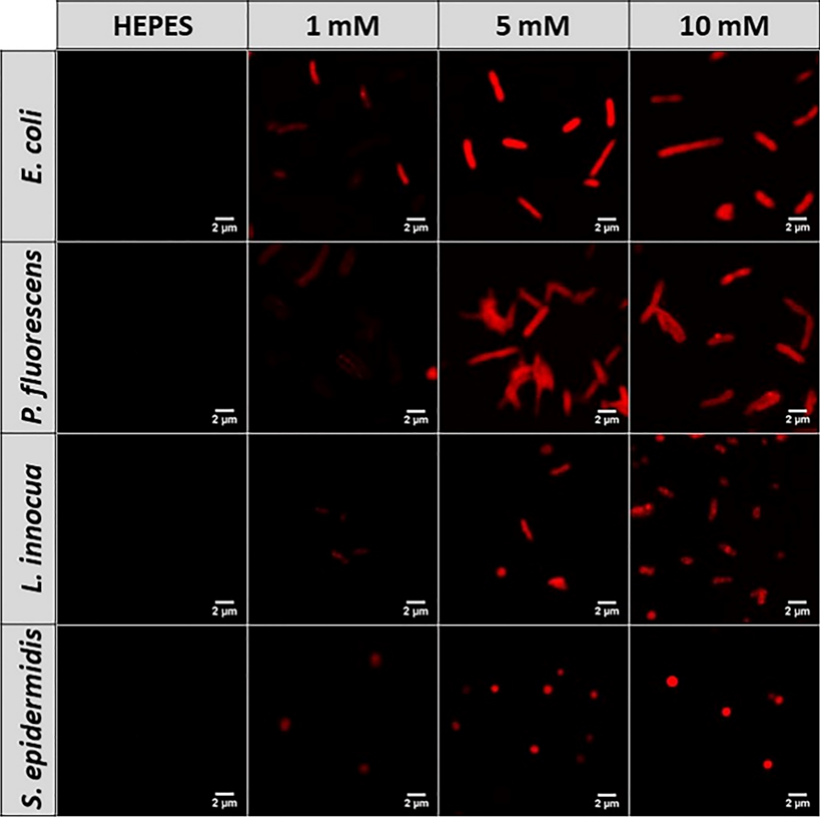
Rh-PE localization in *E. coli*, *P. fluorescens*, *L. innocua* and *S. epidermidis* upon fusion of Rh-LS at 1, 5, and 10 mM with bacterial cell envelopes and imaged by CLSM.

All bacteria exhibited a similar lipid-dose-dependent increase in fluorescence intensity. The fluorescence intensity of the sample and the percentage of stained cells were low at 1 mM DOTAP/DOPE concentration (12% stained cells in *E. coli* and less for the remaining bacteria). When exposed to 5 mM lipids, the fusion efficiency dramatically increased, resulting in 91%, 69%, 79%, and 90% stained cells in *E. coli*, *P. fluorescens*, *L. innocua*, and *S. epidermidis*, respectively. For 10 mM lipids, 100% stained cells were obtained for all bacteria, and the fluorescence intensities also increased. These results clearly demonstrated that the concentration of liposomes plays an important role on fusion efficiency of DOTAP/DOPE/DSPE-PEG liposomes with bacteria. CLSM confirmed the presence of liposomal fluorescently labeled lipids in the bacterial cytosol (Figure 3), while the free Rh-PE lipid was only weakly detected (data not shown). Before visualization, bacteria were also stained with the lipophilic dye SynaptoRed C2 to counterstain the bacterial envelopes; however, our attempts of staining exclusively the phospholipids were hampered by the rapid internalization of this dye when the cells were in the presence of liposomes (Figure S4, Supporting Information). The same was not observed in control bacteria exposed to buffer that showed a fluorescent ring surrounding the Gram-negative bacterial cells. For Gram-positive bacteria, which are smaller in size, this effect is not so apparent. This indicates that cationic liposomes may permeabilize the Gram-negative bacterial envelopes.

### Delivery Efficiency of LipoNAMs

**Figure 4:**
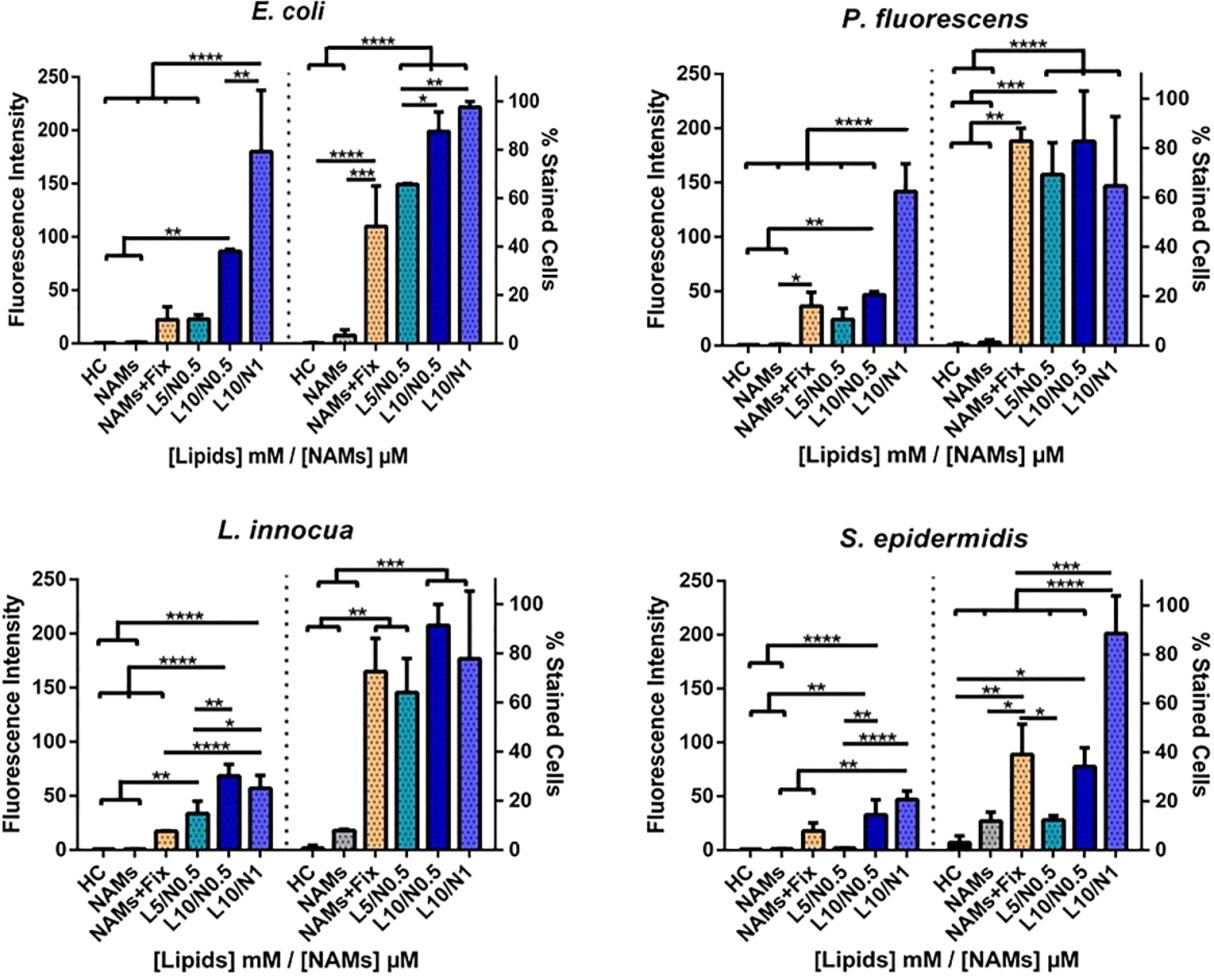
Delivery efficiency of LipoNAMs assessed by FISH in *E. coli*, *P. fluorescens*, *L. innocua*, and *S. epidermidis*: effects of lipid concentration (5 mM vs 10 mM) and concentration of NAMs (0.5 μM vs 1 μM). Fluorescence intensity quantified by flow cytometry and normalized in relation to HC: HC, HEPES control; NAMs, bacteria incubated with 1 μM FAM-labeled NAMs (in HEPES buffer); NAMs+Fix, fixed bacteria incubated with 1 μM FAM-labeled NAMs (in hybridization solution); L5/N0.5, bacteria exposed to 5 mM LipoNAMs containing 0.5 μM FAM-labeled NAMs; L10/N0.5, bacteria exposed to 10 mM LipoNAMs containing 0.5 μM FAM-labeled NAMs; L10/N1, bacteria exposed to 10 mM LipoNAMs containing 1 μM FAM-labeled NAMs. The results are plotted as mean ± standard deviation of three repeated assays. Differences are statistically significant when *P* < 0.05 (*P < 0.05; **P < 0.01; ***P < 0.001; ****P < 0.0001). Brackets and lines indicate the groups being compared.

Based on the high fusion efficiency that was previously obtained for the unloaded liposomes at 5 and 10 mM DOTAP/DOPE lipids, these concentrations were further evaluated for their ability to deliver 6-carboxyfluorescein (FAM)-labeled NAMs in different bacteria by flow cytometry. We simultaneously studied the effect of the contents of lipid and NAMs on the delivery efficiency, by producing 5 mM LipoNAMs with 0.5 μM NAMs (L5/N0.5) and 10 mM LipoNAMs with 0.5 and 1 μM NAMs (L10/N0.5 and L10/N1, respectively). Because nucleic acids are too large to diffuse through the bacterial envelope unassisted, the incubation of naked NAMs with intact bacteria resulted in negligible fluorescence.^15,17,18^ In fact, a positive staining using naked NAMs was only obtained when bacteria were previously chemically fixed/permeabilized. In contrast, LipoNAMs could efficiently internalize NAMs in bacteria, even at a higher extent than chemical fixation/permeabilization, as observed from the higher bacterial fluorescence intensity and percentage of stained cells (Figure 4). However, when considering fluorescence intensity, LipoNAMs appear to deliver FAM-labeled NAMs in Gram-negative bacteria with higher efficiency than in Gram-positive bacteria. As for the effect of lipid concentration, all bacteria presented higher fluorescence intensity when exposed to increasing lipid concentrations (from L5/N0.5 to L10/N0.5) (Figure 4), reflecting the higher internalization of NAMs. This is likely due to the improved fusion at higher lipid concentrations seen in the previous section. Although *S. epidermidis* presented the same uptrend, the delivery efficiency with LipoNAMs (5 mM) loaded with 0.5 μM NAMs (L5/N0.5) was much lower than that for the remaining bacteria. Regarding the influence of the concentration of NAMs on the delivery of LipoNAMs, the fluorescence intensity of the Gram-negative bacteria (*E. coli* and *P. fluorescens*) could be significantly improved when doubling the concentration of NAMs loaded in the LipoNAMs (from L10/N0.5 to L10/N1) (Figure 4). Differently, in the Gram-positive bacteria tested (*L. innocua* and *S. epidermidis*), increasing the concentration of NAMs did not produce any significant change in the fluorescence intensity.

**Figure 5:**
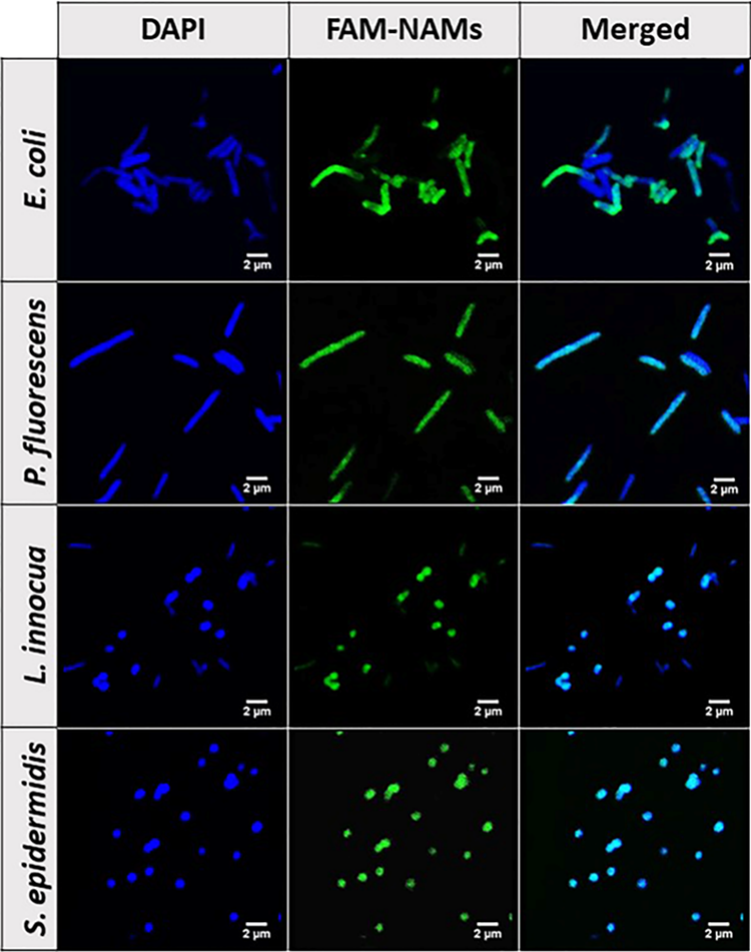
Localization of NAMs in *E. coli*, *P. fluorescens*, *L. innocua*, and *S. epidermidis* cytosol upon delivery by 10 mM LipoNAMs containing 1 μM NAMs (L10/N1) imaged by CLSM: DAPI, bacteria stained with DAPI; FAM-NAMs, FAM-labeled NAM staining; Merged, overlay of DAPI and FAM-NAM images.

The delivery of NAMs in the bacterial cytosol was also confirmed by CLSM. Upon fusion of LipoNAMs (L10/N1), the bacteria were counterstained with DAPI to stain the DNA in the cytoplasm. A colocalization of the staining of DAPI- and FAM-labeled NAMs was observed for all bacteria (Figure 5), meaning that LipoNAMs were indeed able to transport NAMs to the cytosol, where they can bind to the target rRNA. It is also important to notice that not all DAPI-labeled cells are stained with FAM. This observation confirms that even for L10/N1 the percentage of stained cells is not 100%, as also indicated in Figure 4.

### Bacterial Surface Zeta-Potential

Changes in the bacterial envelopes can affect the electrophoretic mobility of bacteria, and therefore, the action of membrane active agents can be detected by zeta-potential measurements.^19,20^ The structural changes on the bacterial envelopes, caused by the fusion of liposomes, were analyzed by measuring the zeta-potential of the samples treated with increasing concentrations of lipids (Figure S5, Supporting Information). The first aspect highlighted by this study was the very low absolute value of the zeta-potential of the *P. fluorescens* envelope in comparison with the remaining bacteria, which is in line with previously reported works.^21^ This low zeta-potential (around −3.0 mV) was kept almost unchanged after exposure to all concentrations of liposomes (Figure S5, Supporting Information). In contrast, *E. coli* control cells exhibited more negative zeta-potential (−39 ± 2 mV) while the Gram-positive bacteria tested had similar mean values around −28 mV. Liposomes at 1 mM lipids produced no shift in the zeta-potential of all bacteria. On the other hand, 5 mM lipids increased 18% the zeta-potential of *E. coli* (in relation to *E. coli* control cells), while they did not significantly affect the overall values in *L. innocua* and *S. epidermidis* (Figure S5, Supporting Information). Differently, the 10 mM concentration similarly affected these three bacteria, resulting in a significant increase in the zeta-potential values of 32%, 30%, and 34% for *E. coli, L. innocua*, and *S. epidermidis*, respectively.

### Release of Intracellular Nucleic Acids

**Figure 6:**
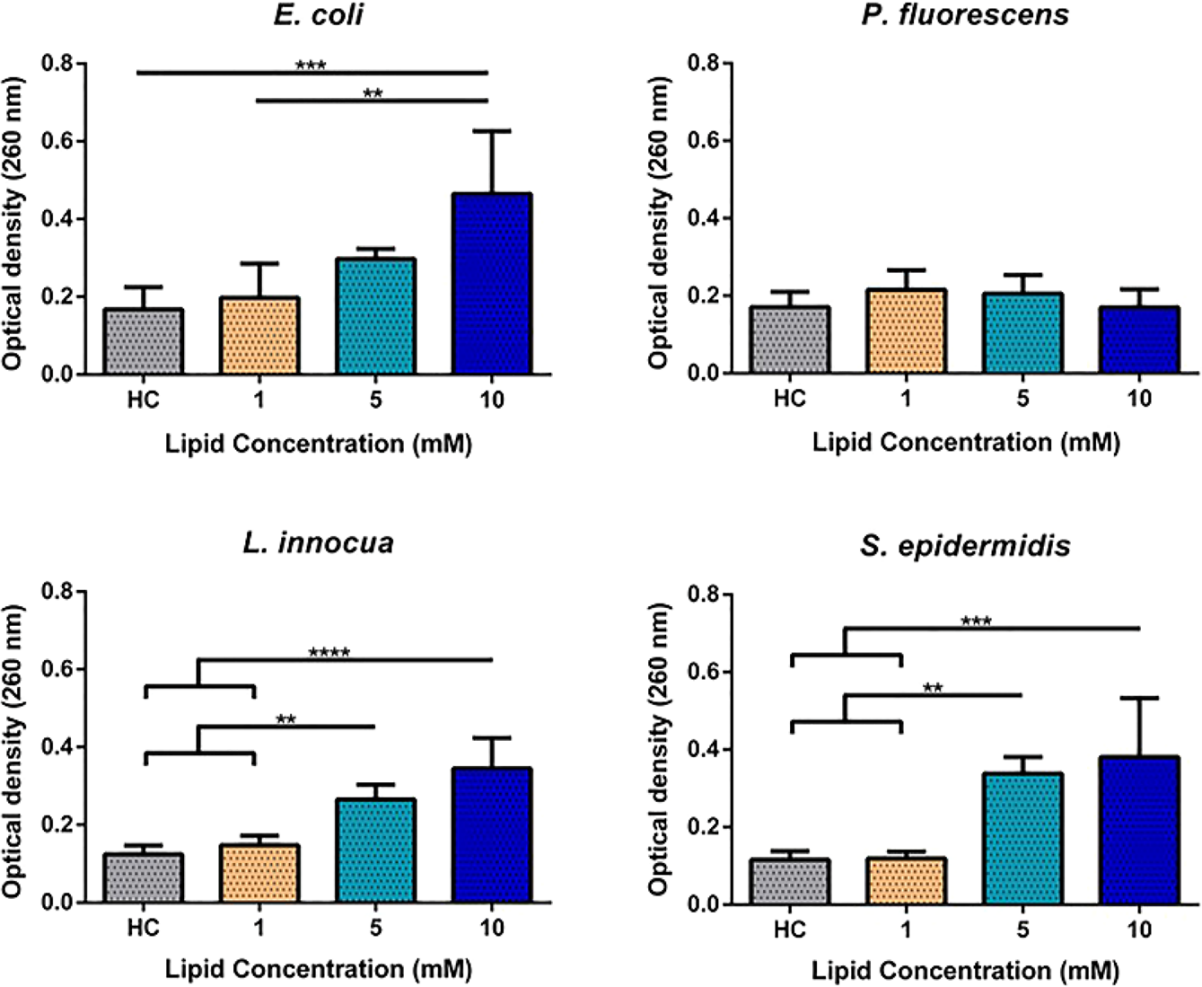
Release of nucleic acids by *E. coli*, *P. fluorescens*, *L. innocua*, and *S. epidermidis* induced by the exposure of bacteria to HEPES (HC, HEPES control) and 1, 5, and 10 mM (lipid concentration) unloaded liposomes (LS). The results are plotted as mean ± standard deviation of five repeated assays. Differences are statistically significant when *P* < 0.05 (*P < 0.05; **P < 0.01; ***P < 0.001; ****P < 0.0001). Brackets and lines indicate the groups being compared.

To evaluate the extension of damage on bacterial envelopes as result of liposome fusion, we also looked for the presence of macromolecules in supernatants upon exposure of bacteria to LS. Specifically, we evaluated the releasing profile of nucleic acids by bacteria (Figure 6). At 1 mM liposomes, the cellular material released by both Gram-positive bacteria followed the profile of the HEPES control whereas in the Gram-negative group only a slight increase was observed. In the presence of 5 mM liposomes, *E. coli* recorded an increase in the release of nucleic acids, reaching the highest value at 10 mM. In contrast, the releasing profile of *P. fluorescens* did not change with the increase of the concentration of liposomes. In Gram-positive bacteria, the release of nucleic acids also increased with the concentration of liposomes, particularly for *S. epidermidis*. Overall, the amount of released nucleic acids increased with the concentration of liposomes, with the effect being more pronounced at 10 mM lipids except for *P. fluorescens*, which did not show significant leakage. The presence of nucleic acids in supernatant indicates a positive effect of the liposomes on the envelope permeability of bacteria. The leakage of macromolecules from cytoplasm also suggests that DOTAP/DOPE/DSPE-PEG liposomes might interact with bacterial envelopes by a pore forming mechanism.

### Morphological and Structural Changes on Bacteria

**Figure 7:**
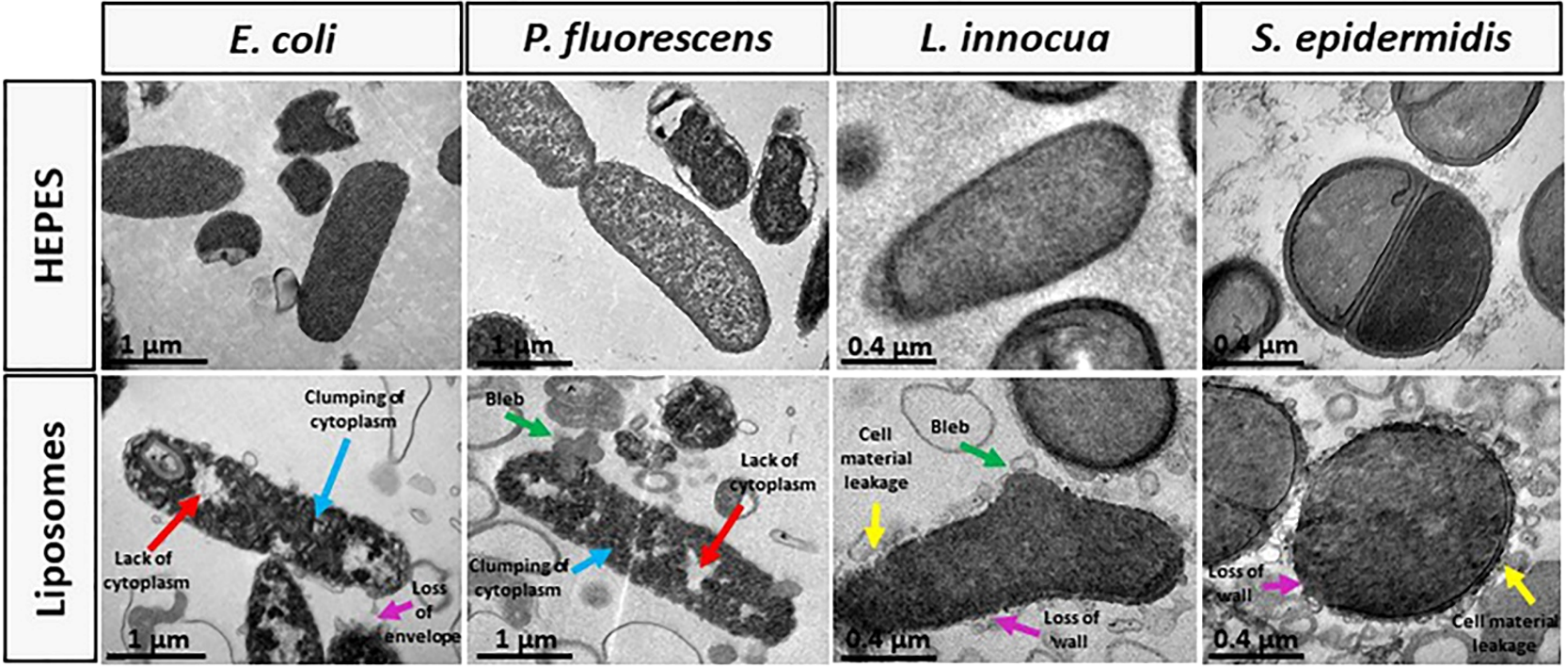
Morphological and structural changes on *E. coli*, *P. fluorescens*, *L. innocua*, and *S. epidermidis* induced by the exposure of bacteria to 10 mM (DOTAP/DOPE concentration) unloaded liposomes (LS) imaged by TEM: HEPES, bacteria incubated in HEPES buffer; Liposomes, bacteria exposed to 10 mM LS; red arrows, lack of cytoplasm; blue arrows, clumping of cytoplasm; pink arrows, loss of envelope/wall; green arrows, bleb; yellow arrows, cell material leakage.

Looking for an explanation for the substantial changes on the bacterial envelope exposed to 10 mM of liposomes, we carried out a structural study of bacteria by TEM (Figure 7). Gram-negative bacteria exposed to 10 mM liposomes exhibited damages envelopes, leakage of cytoplasmic material, and bleb-like structures. Moreover, the bacteria exhibited a heterogeneous distribution and clumping of genetic material in the cytosol. Gram-positive bacteria also presented rupture of the cellular envelope with release of cytosolic contents and bleb-like structures. The leakage of cytoplasmic material was observed for all cells. However, the extension of cell deformation was less dramatic for Gram-positive bacteria, possibly because of a more rigid shape provided by the thicker peptidoglycan wall. Another distinctive aspect, in comparison with Gram-negative bacteria, was the cytoplasm organization which exhibited a more homogeneous distribution of nucleic acids and less evident clumping.

### Viability Assay

Due to the damage and morphological changes observed in all bacteria, we evaluated the effect of the concentration of liposomes on the bacterial viability using the resazurin assay. The viability of bacteria exposed to different concentrations of unloaded liposomes was compared with HEPES control when this reached the maximum resorufin fluorescence intensity.

**Figure 8:**
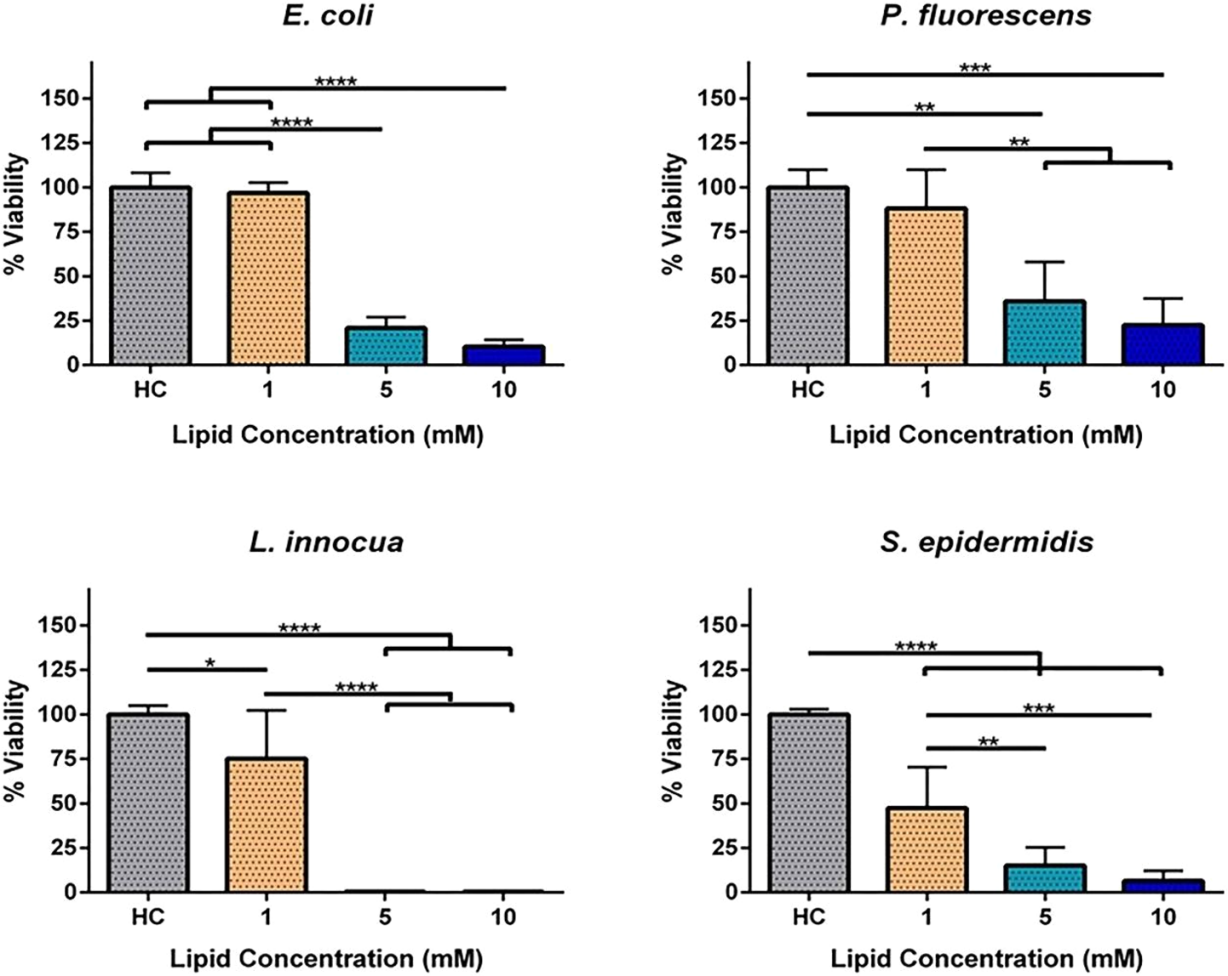
Percentage of viability of *E. coli*, *P. fluorescens*, *L. innocua*, and *S. epidermidis* exposed to HEPES (HC, HEPES control) and 1, 5, and 10 mM unloaded liposomes (LS). Each assay was independently repeated four times. Differences are statistically significant when *P* < 0.05 (*P < 0.05; **P < 0.01; ***P < 0.001; ****P < 0.0001). Brackets and lines indicate the groups being compared.

The viability of all bacteria was affected in a dose-dependent manner (Figure 8). One millimolar liposomes had no effect on the viability of Gram-negative bacteria whereas *L. innocua* and *S. epidermidis* suffered a reductions 25% and 52%, respectively. Five millimolar liposomes reduced the viability of *E. coli*, *P. fluorescens*, and *S. epidermidis* by 79%, 64%, and 85%, respectively, and 10 mM liposomes reduced it by 90%, 76%, and 94%, respectively. *L. innocua* was the most sensitive bacteria to liposome interaction with 99.5% viability reduction at 5 mM liposomes.

These results show that the viability of Gram-negative and Gram-positive bacteria exhibits different susceptibilities to fusogenic cationic liposomes, with the Gram-positive bacteria being significantly more sensitive. It is known that the outer membrane offers Gram-negative bacteria an additional protection against several cationic antimicrobials by preventing access to the inner membrane.^22^ Therefore, the absence of an outer membrane can explain the higher susceptibility of Gram-positive bacteria to cationic lipids.

## Discussion

Understanding the human microbiota ecology is a challenging task but with enormous envisioned value for medical purposes.^2,3^ Host colonization models have been built by (i) imaging transparent animals, such as *Caenorhabditis elegans* and zebrafish,^6,23,24^ (ii) DNA sequencing of laser capture microdissections of *ex vivo* discrete gut regions of mice,^25^ or (iii) mapping of microbes in *ex vivo* organs using confocal microscopy.^26^ However, even animal models expressing human phenotypes are still limited to fully understand the complexity of host–microbe interactions due to the evident distinct anatomical, physiological, or immune features.^27−29^

Alternatively, bacteria can be labeled *in vivo* and visualized by endomicroscopic techniques.^13,30^ Indeed, endoscope-based confocal laser endomicroscopy allows real-time visualization of mucosa with magnification up to 1000-fold, relative high resolution, and laser penetration depth up to 250 μm,^30,31^ allowing diagnosis of bacterial infections and pathologic lesions in bronchoalveolar and gastrointestinal mucosa using off-target contrast agents (e.g., fluorescein and acriflavine).^31−34^

Aiming to improve the understanding of microbiota ecology and diagnosis of infectious diseases, we evaluated FAM-labeled LNA/2′OMe/PS loaded in DOTAP/DOPE/DSPE-PEG liposomes as probes to detect bacteria belonging to the genera *Escherichia*, *Pseudomonas*, *Listeria*, and *Staphylococcus*. Here, our purpose was to characterize the delivery of the LipoNAMs for different bacteria, and as such, a nonspecific probe was used. For the discrimination of the different species of the microbiota, specific probes should be used instead. Due to the extensive characterization of the human microbiome,^2,35^ there are already many genomic sequences available that allow identification of sequences to stain specific bacteria.

In addition, we conducted a comprehensive study of the liposome–bacterium interactions to unveil the delivery mechanism of the LipoNAMs. *E. coli* and *S. epidermidis* were selected as part of the human microbiota. In order to evaluate how the cell shape and the physicochemical properties of different bacterial envelopes could affect the performance of these liposomes, two additional bacterial strains were added to the study. *L. innocua* is a Gram-positive bacterium that exhibits a bacillus shape, differing, therefore, from the coccoid shape of *S. epidermidis*. *P. fluorescens* is a Gram-negative bacterium with a very hydrophobic surface,^21^ being, therefore, a very interesting model to assess the performance of cationic liposomes against hydrophobic bacterial envelopes.

The interaction of Rh-labeled DOTAP/DOPE/DSPE-PEG liposomes was studied by flow cytometry and CLSM, with respect to different lipid concentrations and different bacteria. Both methods showed that the liposomes containing the lowest lipid concentration (1 mM) had a negligible performance in all bacteria, which was likely due to a low liposomes/bacteria ratio and, therefore, a low permeation effect on the bacterial envelopes (Figures 1 and 2). At 5 mM the liposomes–bacteria interaction increased significantly, and at 10 mM the maximum fluorescence intensity and 100% stained cells were reached (Figure 2). The fluorescence intensity increased in the order *L. innocua ≈ S. epidermidis* < *P. fluorescens* < *E. coli*. The phosphatidylethanolamine (PE) content in the bacterial membranes can explain the highest fluorescence intensity observed in *E. coli*, since bacteria with lower PE content in the outer membrane (*Pseudomonas aeruginosa*) or very little PE in the cytoplasmic membrane (*Staphylococcus aureus*) have exhibited lower fusion rates with fusogenic liposomes.^36−38^ Nonetheless, the liposomes showed high interaction efficiency with all bacteria. This broad-spectrum interaction of DOTAP/DOPE/DSPE-PEG liposomes is very interesting, as they may be used to deliver labeled NAMs to different bacteria, without changing the liposomal formulation.

Considering the findings of the fusion assay, we compared the most efficient lipid concentrations (5 and 10 mM) for delivery of NAMs (Figure 4). These LipoNAMs were produced with a loading efficiency between 41 and 51% as assessed by both exclusion chromatography and RiboGreen methods, but additional studies can still be performed to determine the concentration of the lipid nanoparticles and NAMs loaded in each liposome, for instance, using nanoparticle tracking analysis.^39,40^ In accordance with the fusion assay (Rh-LS, Figure 2), the conditions where liposomes–bacteria fusion was higher led to increased NAM delivery efficiencies, as the maximal NAM fluorescence was observed using the highest liposome lipid concentrations and in *E. coli*. Then, it was also tested if the internalization of NAMs could be further enhanced by loading a higher concentration of NAMs in the liposomes. Therefore, 0.5 and 1 μM NAMs (determined by interpolation with the standard curve) were used in LipoNAMs containing 10 mM lipids. The fluorescence intensity indeed increased in the Gram-negative bacteria (Figure 4). Surprisingly, it did not in the Gram-positive bacteria tested, which kept a similar fluorescence intensity with both concentrations of NAMs. One possible explanation may be lower penetration of probes across Gram-positive bacteria. Indeed, and despite the differences in the permeabilization methods, FISH studies employing fixed bacteria have also reported lower fluorescence intensities in Gram-positive bacteria than in Gram-negative bacteria.^41,42^

The fusion mechanism described in the literature^43,44^ for the interaction of fluid liposomes with the Gram-negative bacterial outer membrane assumes that fusion occurs by lipid mixing and merging of the two lipid membranes (liposomes and the bacterial outer membrane). However, Gram-positive bacteria do not have an outer membrane for lipid mixing. Thus, if fusion occurs, it should follow a different mechanism, since the liposomes would need to overcome a thick peptidoglycan layer to fuse with the cytoplasmic membrane underneath.^17,38^ To enlighten the different fusion and delivery mechanisms of DOTAP/DOPE/DSPE-PEG liposomes in both Gram-negative and Gram-positive bacteria, structural and morphological studies were carried out as a function of the liposomal lipid concentration (Figures 5, 6, and S3 of the Supporting Information) and the viability of bacteria monitored upon liposomes–bacteria interaction (Figure 8). *P. fluorescens* showed the highest zeta-potential value remained almost unchanged after exposure to liposomes. However, this does not mean that the surface is not negatively charged. In fact, the zeta-potential of *P. fluorescens* assumed a negative value. This means that negatively charged molecules are still associated with the *P. fluorescens*’ outer membrane allowing the electrostatic interaction with cationic liposomes. To evaluate how the absolute charge of bacteria can affect the interaction with cationic liposomes, additional studies with strains and mutants showing different surface charges need to be conducted. *E. coli* and both Gram-positive positive showed a leakage profile and increase in the zeta-potential that followed the increase in the concentration of the lipids (from 1 to 10 mM), suggesting chemical or structural changes on the envelope surfaces. TEM showed damages in the bacterial envelopes, which are considered the main cause of cell death for the bacteria in contact with 10 mM liposomal lipids. These damages were most likely caused by the action of DOTAP since cationic lipids have shown antimicrobial properties.^45−48^ However, the injuries were more extensive in Gram-negative bacteria which could facilitate the NAMs internalization, thus developing higher fluorescent signals than Gram-positive bacteria (as previously referred). In order to maximize the detection, lipid concentrations of approximately 5 mM should be selected to guarantee a high delivery efficiency and low cell lysis due to the excess of fusion of liposomes. While it would be desirable that the obtained formulation would not decrease the viability of the bacteria, the most important aspect is that the cells can still be visualized with their physical structure and nucleic acid content intact. Both confocal laser scanning microscopy (CLSM) and transmission electron microscopy (TEM) showed that the bacteria preserved their physical structure after exposure to Rh-LS, LipoNAMs, and LS. Therefore, even a liposomal concentration with high antimicrobial activity may allow the detection of bacteria in the organs harboring dense microbial populations such as the large intestine.

**Figure 9:**
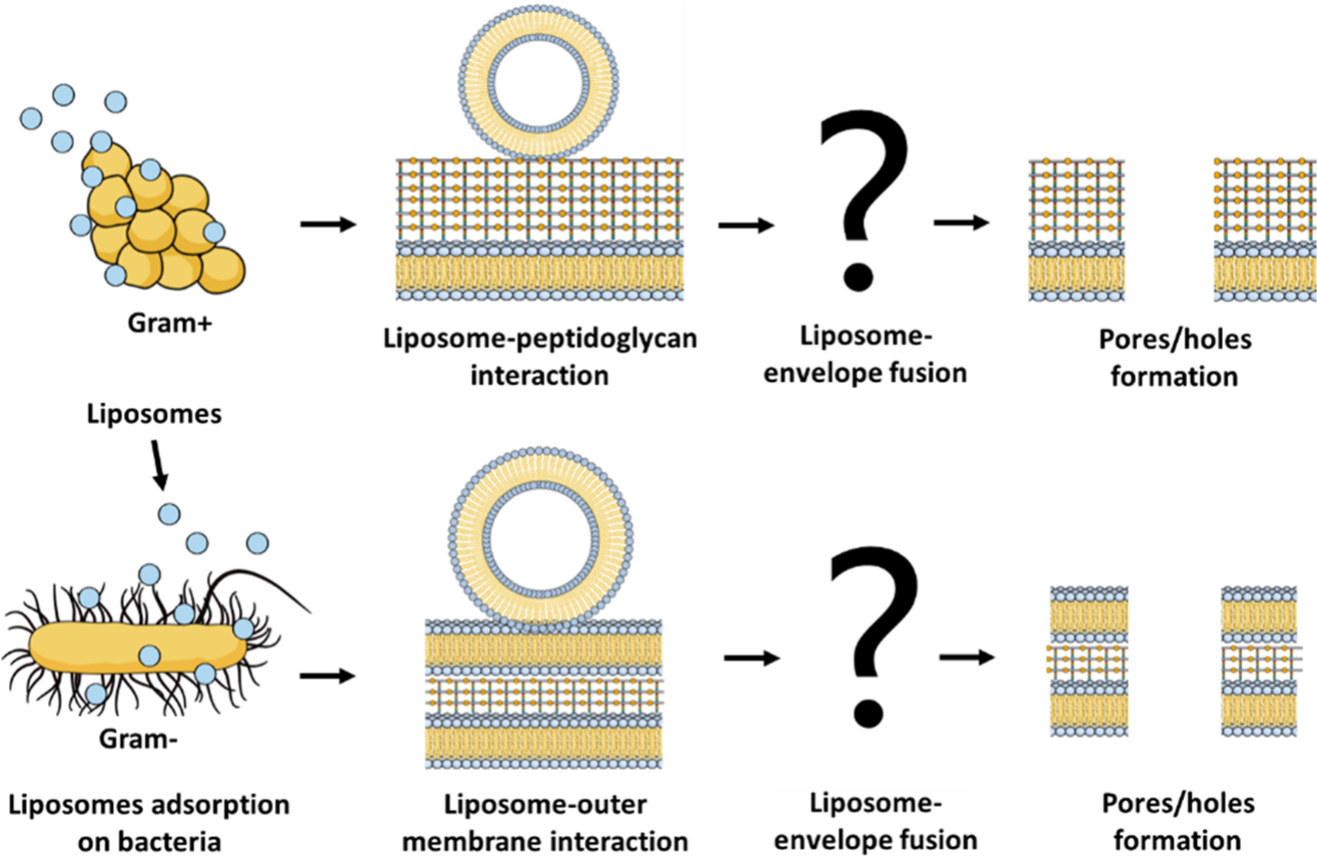
Interaction mechanism of DOTAP/DOPE/DSPE-PEG liposomes with Gram-negative and Gram-positive bacteria for the delivery of the studied NAMs. Gram-negative mechanism: adsorption of liposomes on the bacterial surface; destabilization of the outer phospholipid membrane, peptidoglycan layer, and inner membrane; formation of pores and holes with detachment of patches of the bacterial envelope; leakage of cytoplasmic material. Gram-positive mechanism: adsorption of liposomes on the peptidoglycan layer; rupture of the peptidoglycan; formation of pores with leakage of cytoplasmic material; to a lesser extent, detachment of small patches of the envelope and release of cytoplasmic material. This figure was created with BioRender.com.

Based on our structural and morphological study, we proposed an interaction mechanism for DOTAP/DOPE/DSPE-PEG liposomes with both Gram-negative and Gram-positive bacteria, which is illustrated in Figure 9. In Gram-negative bacteria, the liposomes first interact with the outer membrane, leading to the destabilization of the phospholipid arrangement, followed by destabilization of the peptidoglycan layer and the inner membrane, producing pores. In the presence of high concentrations of liposomes, the high fusion rate can lead to envelope rupture and cell lysis. In Gram-positive bacteria, the liposomes should be able to destabilize the thick peptidoglycan layer upon contact, which leads to the formation of pores and permeabilization of the cytoplasmic membrane. However, the mechanism by which the peptidoglycan is destabilized remains unclear.

This work highlights the potential of LipoNAMs as probes to image and detect bacteria *in vitro*, eliminating time-consuming fixation and permeabilization steps in the traditional FISH protocol. While these are encouraging results, many challenges remain before LipoNAMs can be successfully used to visualize the human microbiota *in vivo*. One is the delivery of LipoNAMs in the gastrointestinal tract that could be addressed using capsules loaded with the lipid vesicles for oral delivery.^49,50^ Future studies should also focus on the optimization of the formulation to overcome toxicity without affecting the uptake of NAMs and testing the oral administration of LipoNAMs loaded in capsules (containing NAMs targeting specific bacteria) to infected mice and bacteria visualization using an *in vivo* confocal endomicroscope.^51^

## Conclusion

Fusogenic cationic liposomes are a promising strategy to deliver nucleic acid probes in bacteria exhibiting different envelope characteristics. However, the fusion and delivery mechanisms of fusogenic cationic liposomes in bacteria are not well understood, which limits the development of efficient formulations. Thus, in this study we developed a comprehensive study of the interaction of such liposomes with both Gram-negative and Gram-positive bacteria, giving special attention to the influence of the lipid concentration. We found that the fusion of LipoNAMs and delivery efficiencies are dependent on the lipid dose and the bacterial envelope type. Moreover, we observed that, at high lipid concentrations, liposomes produced disruption of bacterial envelopes and cells lysis, an effect more pronounced in Gram-negative bacteria. Nonetheless, the liposomes showed high interaction efficiency with all bacteria and broad-spectrum activity. LipoNAMs exhibited interesting features to apply in the diagnosis of bacterial infection using an *in vitro* technique, whose further investigation may allow their application as specific probes to image bacteria *in vivo*, either for research or diagnostic purposes. Future studies will focus on testing the administration of LipoNAMs (containing NAMs targeting specific bacteria) to infected mice and bacteria visualization using *in vivo* confocal endomicroscopy.

## Methods

### NAMs, Lipids, and Staining Reagents

The NAMs used in this work consisted of a 6-carboxyfluorescein-labeled 14-mer oligonucleotide formed by an LNA, 2′OMe, and PS linkage (Eurogentec, Seraing, Belgium). This sequence was designed to target a conserved region of the 16S rRNA in the domain Eubacteria.^52^ The synthetic phospholipids 1,2-dioleoyl-3-trimethylammonium-propane (DOTAP, chloride salt), 1,2-dioleoyl-*sn*-glycero-3-phosphoethanolamine (DOPE, chloride salt), 1,2-distearoyl-*sn*-glycero-3-phosphoethanolamine-*N*-[methoxy(polyethylene glycol)-2000] (DSPE-PEG, ammonium salt), and 1,2-dipalmitoyl-*sn*-glycero-3-phosphoethanolamine-*N*-(lissamine rhodamine B sulfonyl) (Rh-PE) were purchased from Avanti Polar Lipids (Alabaster, AL, USA). 4′,6-Diamidine-2′-phenylindole dihydrochloride (DAPI) was purchased from Sigma-Aldrich (Lisbon, Portugal). All other reagents were of analytical grade.

### Preparation of Liposomes and LipoNAMs

Liposomes were prepared with DOTAP, DOPE, and DSPE-PEG at a molar ratio of 49.5:49.5:1. Loading of the liposomes with the NAMs to produce LipoNAMs was carried out following the ethanol dilution method described by Jeffs et al.^50^ with some modifications (Figure S1, Supporting Information). Briefly, lipids dissolved in chloroform were mixed, dried to form a lipid film by evaporation under a nitrogen stream to avoid oxidation of lipids, and redissolved in 90% (v/v) ethanol to a final concentration of 40.4 mM (Figure S1a). NAMs at 5 μM or 10 μM were prepared in HEPES buffer (HEPES, 10 mM HEPES hemisodium salt, pH = 7.4, Sigma-Aldrich, Lisbon, Portugal) prewarmed at 37 °C for 15 min (Figure S1b). Equal volumes of lipid and NAM solutions were mixed using a multichannel pipet, slightly stirred for 1 min, and incubated at room temperature for 5 min (Figure S1c). The liposomal suspension was diluted in an equal volume of prewarmed HEPES (37 °C) followed by incubation at 37 °C for 15 min to stabilize the lipid vesicles (Figure S1d). The final total lipid content in the PEGylated LipoNAMs was 10.1 mM (7.51 mg/mL). Free NAMs were removed by passing vesicles through a PD SpinTrap G-25 column (GE Healthcare, Pittsburgh, PA, USA) (Figure S1f). The loading efficiency (%LE) was calculated according to eq 1. Briefly, the fluorescence of the LipoNAMs was measured using a microtiter plate reader FLUOstar OMEGA (BMG Labtech, Ortenberg, Germany) equipped with filters ex485-12/em520-10 (gain 800), before (Figure S1e) and after (Figure S1g) removal of free NAMs. The respective concentrations of NAMs were calculated based on a standard curve (*R*^2^ > 99%) constructed for concentrations of FAM-labeled NAMs ranging from 0 to 20 μM.
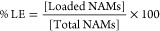
1where [Loaded NAMs] and [Total NAMs] refer to the concentrations of NAMs after and before removal of free NAMs, respectively.

Additionally, encapsulation of NAMs in liposomes was studied using the membrane-impermeable fluorescent dye RiboGreen (Quant-iT RiboGreen RNA Reagent and Kit, Molecular Probes, Eugene, OR, USA) as described by Walsh et al.^51^ with some modifications. Briefly, LipoNAMs were diluted in TE buffer or 0.5% Triton-X100 buffer to lyse liposomes and incubated at 37 °C for 30 min. RiboGreen solution was mixed with either intact or lysed liposomes and incubated at 37 °C for 15 min. The fluorescence intensity was measured using a microtiter plate reader FLUOstar OMEGA (BMG Labtech, Ortenberg, Germany) equipped with filters ex485-12/em530-10 (gain 1700). The content of NAMs was calculated through interpolation with the standard curves of RiboGreen and different concentrations of FAM-labeled NAMs prepared in either TE or Triton-X100 buffers. The %LE was calculated using eq 2:
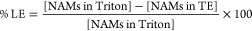
2where [NAMs in TE] and [NAMs in Triton] refer to the concentrations of NAMs in LipoNAMs diluted in TE buffer and LipoNAMs lysed in Triton-X100 buffer, respectively.

LipoNAMs were subsequently purified by the removal of the ethanol used in the preparation of the liposomal suspension, by dilution in HEPES, followed by ultrafiltration using an Amicon ultra-0.5 centrifugal filter device (Merck Millipore, Burlington, MA, USA) (Figure S1h).

Purified LipoNAMs prepared with 5 μM NAMs were then diluted to form a suspension with 10 mM DOTAP/DOPE lipids containing 0.5 μM NAMs (L10/N0.5). Purified LipoNAMs prepared with 10 μM NAMs were diluted to 5 and 10 mM DOTAP/DOPE lipids containing, respectively, 0.5 and 1 μM NAMs (L5/N0.5 and L10/N1, respectively). Unloaded PEGylated liposomes (LS) were produced by mixing the ethanolic lipid solution with prewarmed HEPES. Unloaded PEGylated rhodamine-labeled liposomes (Rh-LS) were prepared by the addition of 2 mM, 0.4 mM, or 0.2 mM Rh-PE to the lipid mixture and dilution of the purified Rh-LS in HEPES buffer to a final DOTAP/DOPE concentration of 1, 5, and 10 mM, respectively, thereby obtaining equal Rh-PE concentrations (0.05 mM). In Figure S2 (Supporting Information) a schematic representation of the Rh-LS production in shown. All the formulations were stored at 4 °C overnight. The average hydrodynamic diameter, polydispersion index (PDI), and zeta-potential of the unloaded liposomes and LipoNAMs were routinely checked by dynamic light scattering (DLS, Figure S1i) using a Malvern ZetaSizer instrument (Malvern Instruments Ltd., Malvern, UK).

### Culture of Bacterial Strains

Gram-negative *Escherichia coli* CSH36 (*E. coli*, Coli Genetic Stock Center, Yale University, CT, USA) and *Pseudomonas fluorescens* ATCC 13525 (*P. fluorescens*, American Type Culture Collection, VA, USA) and Gram-positive *Listeria innocua* CECT 910 (*L. innocua*, Spanish Type Culture Collection, Spain) and *Staphylococcus epidermidis* ATCC 35984 (*S. epidermidis*, American Type Culture Collection, VA, USA) were used in this study. The bacteria were grown for 16–18 h in Tryptic Soy Agar (TSA) and subcultured every week up to five passages. In each assay, bacteria were harvested from TSA plates, inoculated into 10 mL of Tryptic Soy Broth (TSB, Oxoid, Basingstoke, UK), and incubated for 16–18 h at the optimum growth temperature (30 °C for *P. fluorescens* and 37 °C for the remaining bacteria), at 180 rpm. Then, the cultures were diluted in TSB to an optical density of 0.1 at 600 nm (OD_600_) and grown up to the exponential phase, at the previously described conditions. The final bacterial concentration was set to OD_600_ = 0.15 (*P. fluorescens*) or OD_600_ = 0.1 (remaining bacteria).

### Interaction of Liposomes with Bacteria

Bacteria were exposed to liposomes following the experimental procedure described by Santos et al.^15^ with adaptations. Freshly grown bacteria (1 mL) were centrifuged at 16800*g* for 10 min, resuspended in 50 μL of liposomes, and incubated at 37 °C for 1 h. Liposomes were diluted in prewarmed (37 °C) HEPES buffer to a final DOTAP/DOPE concentration of 1, 5, and 10 mM containing 50 μM Rh-PE each. Bacteria incubated in HEPES, at the same conditions, were used as a negative control for autofluorescence. 50 μM liposome-free Rh-PE was also used as control. Then, bacteria were pelleted by centrifugation at 8600*g* for 5 min and rinsed with 500 μL of prewarmed (37 °C) washing solution (15 mM NaCl, 0.1% (v/v) Triton-X, 5 mM Tris base, pH = 10) for 15 min, at 37 °C, to remove liposomes adsorbed on the bacterial surface. Samples were pelleted again by centrifugation at 8600*g* for 5 min. Pellets were resuspended in 500 μL of sterile milli-Q water and sonicated in a bath sonicator at room temperature for 10 min to disperse bacterial cells. Interaction of rhodamine-labeled liposomes (Rh-LS) with bacteria was assessed by flow cytometry and confocal laser scanning microscopy (CLSM) as described in the Supporting Information.

### Fluorescence *In Situ* Hybridization (FISH) in Fixed/Permeabilized Bacteria and with LipoNAMs

FISH was performed as described by Fontenete et al.^52^ with some modifications. Bacteria were prepared as described in the previous section, and 1 mL of each culture was centrifuged at 16800*g* for 10 min followed by bacteria fixation by pellet resuspension in 4% formaldehyde in PBS and incubation for 1 h at room temperature. Bacteria were centrifuged at 8600*g* for 5 min, and Gram-positive bacteria were permeabilized with 4 mg/mL lysozyme (in PBS) for 10 min at room temperature followed by centrifugation. Fixed bacteria were exposed to 1.2 μM naked NAMs in hybridization solution (900 mM NaCl, 20% (v/v) formamide, 5 mM disodium EDTA, 0.1% (v/v) Triton X-100, 50 mM Tris-HCl, pH 7.5) for 1 h at 37 °C, pelleted at 8600*g* for 5 min, and rinsed with 500 μL of prewarmed (37 °C) washing solution (15 mM NaCl, 0.1% (v/v) Triton-X, 5 mM Tris base, pH = 10) for 15 min, at 37 °C. Lastly, samples were pelleted at 8600*g* for 5 min, resuspended in 500 μL of sterile milli-Q water, and sonicated in a bath sonicator at room temperature for 10 min to disperse bacterial cells.

For FISH with LipoNAMs, the bacteria were not fixed but instead exposed to the LipoNAMs following the procedure described in the previous section. LipoNAMs were tested by varying the lipid and NAMs concentrations: lipid content at 5 mM and 10 mM (DOTAP/DOPE concentration) containing 0.5 μM NAMs (L5/N0.5 and L10/N0.5, respectively) and NAMs at 0.5 and 1 μM with 10 mM DOTAP/DOPE content (L10/N0.5 and L10/N1, respectively). Bacteria incubated in HEPES and in 1.2 μM naked NAMs (in HEPES) were used as controls. Delivery of NAMs into the bacteria by the LipoNAMs was quantified via flow cytometry and observed using CLSM as described in the Supporting Information.

### Effect of Liposomes on the Bacterial Envelope Integrity and Morphology

To detect the activity of membrane active agents, bacteria were exposed to LS and the zeta-potential was measured afterward. Briefly, 1 mL of overnight grown bacteria was centrifuged at 16800*g* for 10 min and incubated with LS (50 μL) at DOTAP/DOPE concentrations of 1, 5, and 10 mM for 1 h, at 37 °C. Bacteria were pelleted by centrifugation at 8600*g* for 5 min to remove the supernatant and rinsed with HEPES. Then, bacteria were pelleted again at 8600*g* for 5 min and resuspended in 1 mL of HEPES. The samples were transferred to a zeta-cell DTS1070 for zeta-potential measurements, using a Malvern ZetaSizer instrument (Malvern Instruments Ltd., Malvern, UK).

Release of nucleic acids from the bacterial cells was quantified by measuring the optical density (260 nm) of the supernatant upon interaction of LS with bacteria. The supernatants were diluted in 1200 μL of HEPES and transferred to 1 mL quartz cuvettes to measure the optical density using a spectrophotometer T80 UV/vis spectrometer (PG Instruments Ltd., Leicestershire, UK). As a control for 100% rupture of the bacterial envelope, bacteria were lysed using a tip sonicator applying three pulses ON for 30 s and a 30 s pulse OFF (cycle 0; power 30%; SONOPULS Ultrasonic homogenizers HD 2200, BANDELIN, Germany).^53^

The potential structural changes caused by the 10 mM liposomes on the bacterial envelope were evaluated by transmission electron microscopy (TEM). Visualization was performed at 80 kV (JEOL JEM 1400 microscope, Japan), and digital images were acquired using a CCD digital camera Orious 1100 W (Tokyo, Japan). Bacteria in HEPES were used as control of intact bacteria. The detailed procedure is described in the Supporting Information.

### Viability Assay

The viability of bacteria exposed to HEPES or LS at DOTAP/DOPE concentrations of 1, 5, and 10 mM was assayed by measuring the conversion of nonfluorescent resazurin dye to the pink fluorescent resorufin product^54^ (Supporting Information). Briefly, 1 mL of fresh grown bacteria was centrifuged at 16800*g* for 10 min and exposed to 50 μL of LS at 1, 5, and 10 mM for 1 h, at 37 °C. Bacteria in HEPES were used as control. Bacteria were pelleted by centrifugation at 8600*g* for 5 min and resuspended in 1 mL of TSB, of which 190 μL were transferred (in triplicate) to an untreated 96-well microtiter plate (Orange Scientific, Braine-l’Alleud, Belgium) and mixed with 10 μL of 0.4 mM resazurin (Sigma-Aldrich, St. Louis, MO, USA) solution. Sterile TSB was used as sterility control, and its value was subtracted to each condition. The resorufin fluorescent signal was recorded at each 5 min for 12 h using a microtiter plate reader FLUOstar OMEGA (BMG Labtech, Ortenberg, Germany) equipped with filters ex560-10/em600-10 (gain 800) at 30 °C for *P. fluorescens* or 37 °C for the remaining bacteria. Each assay was independently repeated four times. The percentage of viability was calculated using eq 3:
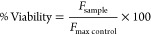
3where *F*_sample_ is the resorufin fluorescence intensity of each sample when the HEPES control reached the maximum fluorescence intensity and *F*_max control_ is the maximum resorufin fluorescence intensity of HEPES used as control. In Figure S3 (Supporting Information) an example of the application of eq 3 is shown.

### Statistical Analysis

Statistical analysis was performed using GraphPad Prism6 software (GraphPad Software, San Diego, CA, USA). The loading efficiencies of LipoNAMs were compared by *t-*test. The hydrodynamic characteristics of LipoNAMs as well as data from the assays containing bacteria were analyzed by one-way analysis of variance (ANOVA) and posthoc Tukey multiple-comparisons test. Results are expressed as mean ± standard deviation of three to five independent assays. Differences are statistically significant when *P* < 0.05 (*P < 0.05; **P < 0.01; ***P < 0.001; ****P < 0.0001).

## Supporting Information Available

Preparation of liposomes and LipoNAMs; DLS procedure; flow cytometry and CLSM analysis; TEM procedure; application of eq 3 in the viability assay; characterization of the lipid vesicles by DLS; graphic of the bacterial surface zeta-potential (PDF)

## Abbreviations

%LE: loading efficiency
2′OMe: 2′-*O*-methyl RNA
CLSM: confocal laser scanning microscopy
DAPI: 4′,6-diamidine-2′-phenylindole dihydrochloride
DNA: deoxyribonucleic acid
DOPE: 1,2-dioleoyl-*sn*-glycero-3-phosphoethanolamine
DOTAP: 1,2-dioleoyl-3-trimethylammonium-propane
DSPE-PEG: 1,2-distearoyl-*sn*-glycero-3-phosphoethanolamine-*N*-[methoxy(polyethylene glycol)-2000]
*E. coli*: *Escherichia coli*
FAM: 6-carboxyfluorescein
FISH: fluorescence *in situ* hybridization
HEPES: HEPES buffer
*L. innocua*: *Listeria innocua*
L5/N0.5: LipoNAMs at 5 mM (DOTAP/DOPE concentration) loaded with 0.5 μM
L10/N0.5: LipoNAMs at 10 mM (DOTAP/DOPE concentration) loaded with 0.5 μM
L10/N1: LipoNAMs at 10 mM (DOTAP/DOPE concentration) loaded with 1 μM
LipoNAMs: liposome-loaded nucleic acid mimics
LNA: locked nucleic acid
LS: unloaded PEGylated liposomes
NAMs: nucleic acid mimics
PDI: polydispersion index
PEG: polyethylene glycol
*P. fluorescens*: *Pseudomonas fluorescens*
PS: phosphorothioate
Rh-LS: unloaded PEGylated rhodamine-labeled liposomes
Rh-PE: 1,2-dipalmitoyl-*sn*-glycero-3-phosphoethanolamine-*N*-(lissamine rhodamine B sulfonyl)
RNA: ribonucleic acid
TEM: transmission electron microscopy
