# Supplementary material for: Liposome Delivery of Nucleic Acids in Bacteria: Toward In Vivo Labeling of Human Microbiota
Source: ACS Infect Dis. 2022 Jun 23;8(7):1218–30. doi: 10.1021/acsinfecdis.1c00601 (PMC9775462; doi:10.1021/acsinfecdis.1c00601)
Supplement: Supplementary file 1 — id1c00601_si_001.pdf [file id1c00601_si_001.pdf]

SUPPORTING INFORMATION

Liposomes delivery of nucleic acids in bacteria: Towards *in vivo* labelling of human microbiota

Luís Moreira[a,b], Nuno M Guimarães[a,b *], Sara Pereira[a,b], Rita S Santos[a,b], Joana A Loureiro[a,b], Maria C Pereira[a,b], Nuno F Azevedo[a,b]

[a]*LEPABE - Laboratory for Process Engineering, Environment, Biotechnology and Energy, Faculty of Engineering, University of Porto, Rua Dr. Roberto Frias, 4200-465 Porto, Portugal*
[b]*ALiCE - Associate Laboratory in Chemical Engineering, Faculty of Engineering, University of Porto, Rua Dr. Roberto Frias, 4200-465 Porto, Portugal*

Corresponding author: Nuno M Guimarães
E-mail address: nguimaraes@fe.up.pt

Material and Methods

Preparation of liposomes and LipoNAMs

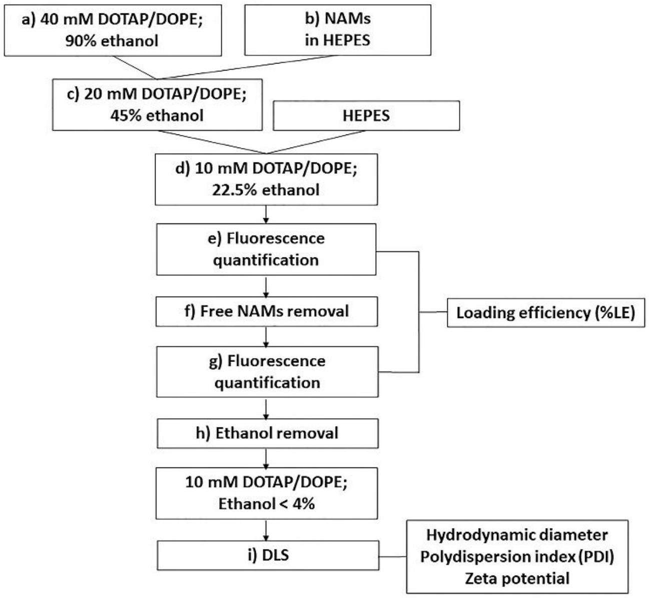

Figure S1. Schematic representation of liposomes production and purification protocol. The concentrations of the DSPE-PEG and Rh-PE lipids were omitted to simplify the scheme representation.

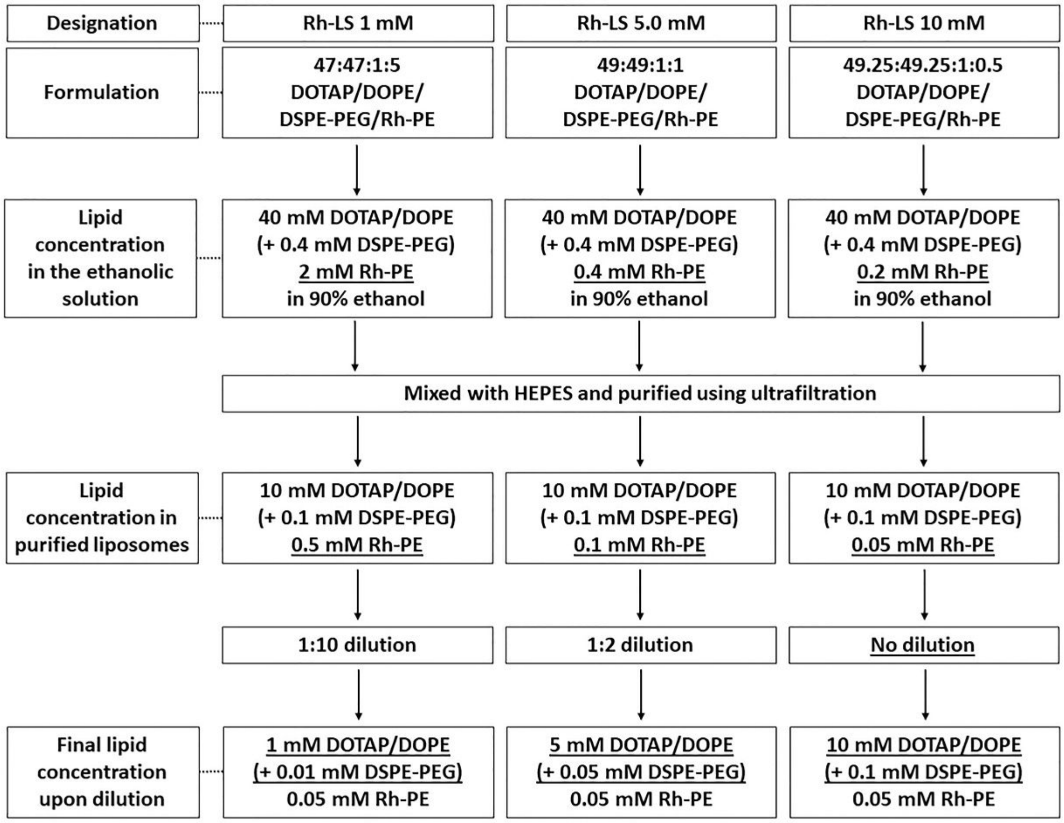

Figure S2. Schematic representation of Rh-LS production. The concentration of DOTAP, DOPE and DSPE-PEG in the ethanolic solution was kept constant between formulation, only varying Rh-PE concentration. After dilution, Rh-LS suspensions consisted of different concentrations of DOTAP, DOPE and DSPE-PEG but containing the same Rh-PE concentration (0.05 mM). As all diluted Rh-LS suspensions had the same concentration of Rh-PE lipid, differences in the fluorescence intensity and percentage of stained cells (determined by flow cytometry) are only due to the differences in the DOTAP, DOPE and DSPE-PEG concentrations. Underlying words represent the lipid concentration that differ between samples each step.

Liposomes characterization by Dynamic Light Scattering

Hydrodynamic diameter, polydispersion index (PDI) and zeta-potential were determined at 25 °C by Dynamic Light Scattering (DLS) using a Malvern ZetaSizer instrument (Malvern Instruments Ltd., Malvern, UK) with a 173° non-invasive backscatter (NIBS) angle. 10 µL microliters of liposomes suspensions were diluted in 900 µL of HEPES and transferred to a disposable cuvette for hydrodynamic diameter and PDI analysis applying the Mark-Houwink model. Three measurements with 10 runs each were done. Then, the

diluted suspension was transferred to a zeta-cell DTS1070 for zeta-potential analysis applying the Smoluchowski model. Each value corresponds to 3 measurements with 10 runs each.

Flow Cytometry analysis

The flow cytometry analysis was carried out at the Translational Cytometry facility, i3S, University of Porto, Portugal. Before cytometric analysis of the bacteria, the bath sonicated samples were filtered through a 10 µm pore size filter (CellTrics®, Görliz, Germany). FAM fluorescence intensity was quantified by a BD FACSCalibur™ flow cytometer (BD Biosciences, San Jose, CA, USA) equipped with a 488 nm argon ion laser. Rhodamine fluorescence was analyzed with a BD FACSARIA II (BD Biosciences, San Jose, CA, USA) equipped with a 561 nm laser and PE-Texas Red® filter. Data were analyzed with FlowJo v10 software (FlowJo, LLC, Ashland, OR, USA). Forward angle light scatter (FS), side angle light scatter (SS), green (FL1) or red (Texas-Red) fluorescence were detected at logarithmic scale. For each sample, a minimum of 50000 events were collected. Data in triplicate were normalized to the autofluorescence control (bacteria in HEPES buffer) and plotted as mean ± standard deviation of arbitrary fluorescence units (AFU).

Confocal Laser Scanning Microscopy (CLSM)

In order to immobilize bacteria for visualization, glass slides (VWR, Portugal) were coated with 0.1% (w/v) poly-L-lysine (Sigma-Aldrich, Lisbon, Portugal) and incubated 30 min at room temperature in a humidified chamber. The excess of poly-L-lysine solution was removed, and slides dried at room temperature. The concentration of bacteria suspensions was set at 0.3 (OD_{600}) and 20 µL placed on the poly-L-lysine coating for immobilization during 1h, at room temperature in a humidified chamber. Non-adherent bacterial cells were washed out with milli-Q water and the slides dried at 37 °C. The samples were mounted with one drop of non-fluorescent immersion oil for visualization.

Bacteria exposed to Rh-LS and immobilized on slides were stained with 15 µL of 5 µg/mL SynaptoRed C2 (Sigma-Aldrich, Lisbon, Portugal) in Hank's Balanced Salt Solution (HBSS) and incubated from 3 min (Gram-negative) to 5 min (Gram-positive), at room temperature. After removal of SynaptoRed C2 excess, the samples were mounted with one drop of non-fluorescent immersion oil.

Bacteria exposed to LipoNAMs L10/N1 were counterstained with DAPI to stain the bacterial cytoplasm. Bacteria were incubated with 50 µL of 0.01% (w/v) DAPI for 10 min at room temperature, pelleted by centrifuged at 8600 x g for 5 min and washed with pre-warmed (37 °C) washing solution (15 mM NaCl, 0,1% (v/v) Triton-X, 5 mM Tris Base, pH=10) for 15 min, at 37 °C. Bacteria were pelleted at 8600 x g for 5

min, resuspended in sterile milli-Q water and sonicated in a bath sonicator at room temperature for 10 min before immobilization on the coated glass slides.

All samples were visualized at the Advanced Light Microscopy facility, i3S, University of Porto, Portugal on a Leica TCS SP5 Confocal Laser Scanning Microscope (Leica Microsystems, Wetzlar, Germany) equipped with an objective HC PL APO 63x /1.40 Oil, lasers 405 nm, 488 nm, 514 nm and 561 nm and filters PMT 415-481 nm, PMT 498-602 nm, PMT 784-800 and HyD 568-622 nm for visualization of DAPI, FAM, SynaptoRed C2 and Rhodamine staining, respectively. For each region of interest 8-14 serial optical sections (z-sections) from bottom to top were collected. Images of middle sections were analyzed using the Figi (ImageJ, open platform for scientific image analysis) software. Corrections for brightness and contrast were performed in order to improve the quality of the merged picture.

Transmission Electron Microscopy (TEM)

The potential structural changes caused by the liposomes on the bacterial envelope was evaluated by TEM. 1 mL of fresh grown bacteria was centrifuged at 16800 x g for 10 min and resuspended in 50 µL of HEPES or 10 mM LS and incubated for 1 h, at 37 °C. Samples were pelleted by centrifugation at 8600 x g for 5 min and fixed with Karnovsky's fixative with sucrose (2% glutaraldehyde, 3% paraformaldehyde, 5% sucrose, 0.1 M sodium cacodylate buffer, pH 7.4) for 2 h, at room temperature, and then stored at 4 °C, overnight. The samples were then delivered to Histology and Electron Microscopy Service (HEMS) at i3S, University of Porto, Portugal where they were processed for TEM analysis. Briefly, samples were washed in 0.1 M sodium cacodylate buffer and fixed in 2 % osmium tetroxide in the 0.1 M sodium cacodylate buffer overnight, followed by staining with 1 % uranyl acetate overnight. After inclusion in Histogel™ (Thermo, HG-4000-012), dehydration of samples was carried out in 50%, 70%, 80% and 100% ethanol (three times) and pure propylene oxide (three times), for 10 min each. The samples were then embedded in EPON resin by a gradient of EPON solutions in propylene oxide (1:3, 1:1 and 3:1 for 2 h each and only EPON for 48 h). When required, the samples were pelleted by centrifugation at 4200 x g for 5 min. Sections with 60 nm thickness were prepared on a RMC Ultramicrotome (PowerTome, USA) using a diamond knife and recovered to 200 mesh Formvar Ni-grids, followed by immersion in 2% uranyl acetate and saturated lead citrate solutions for 7 min each. Visualization was performed at 80 kV (JEOL JEM 1400 microscope, Japan) and digital images were acquired using a CCD digital camera Orious 1100 W (Tokyo, Japan).

Viability assay

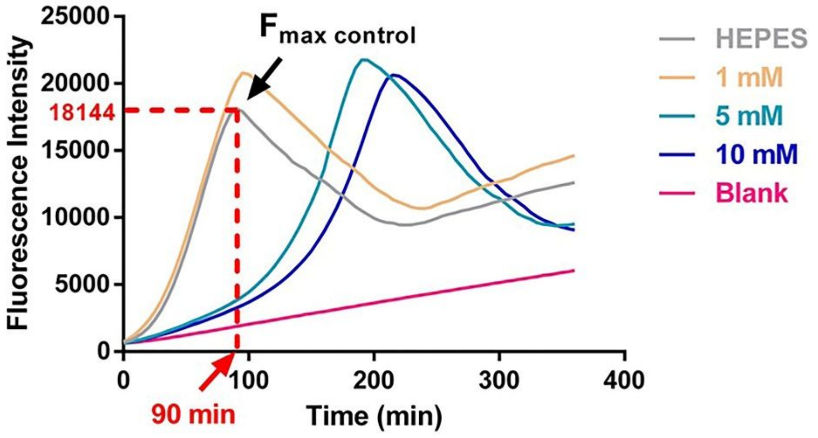

Sample	Fluorescence (at 90 min)
F HEPES	18144
F 1mM	20157
F 5mM	3816
F 10mM	3260
F Blank	1884

Figure S3. Graphical representation of a viability test using the resazurin assay. At 90 min, the HEPES control reached its maximum resorufin fluorescence ($F_{HEPES} = F_{max\ control}$). At this time (90 min), the fluorescence of the blank (F_{Blank}) was subtracted to the value of each sample (F_{HEPES}, F_{1mM}, F_{5mM}, and F_{10mM}). The values of F_{1mM}, F_{5mM}, and F_{10mM} samples were then divided by the F_{HEPES} value and multiplied by 100 to calculate the percentage of viability (Equation 2).

Results

Characterization of unloaded liposomes and LipoNAMs by DLS

Table S1. Comparison of hydrodynamic diameter, PDI and zeta-potential of unloaded PEGylated liposomes (LS), LipoNAMs at 10 mM (DOTAP/DOPE concentration) loaded with 0.5 µM (LN10/0.5) and 1 µM (LN10/1) of NAMs and unloaded PEGylated rhodamine-labelled liposomes (Rh-LS) at 1, 5 and 10 mM (DOTAP/DOPE concentration) containing 50 µM of Rh-PE lipid. Loading efficiency (%LE) of 10 mM (lipid concentration) LipoNAMs containing 0.5 µM (LN10/0.5) and 1 µM (LN10/1) of NAMs.

Liposomes designation	Formulation	Diameter (nm)	PDI	Zeta-potential (mV)	%LE (E1)[a]	%LE (E2)[b]
**LS**	49.5:49.5:1 DOTAP/DOPE/ DSPE-PEG	123 ± 14	0.20 ± 0.03	38 ± 4	n/a[c]	n/a[c]
**L10/N0.5**	49.5:49.5:1 DOTAP/DOPE/ DSPE-PEG + NAMs	107 ± 31	0.21 ± 0.03	36 ± 5	43 ± 11	46 ± 5
**L10/N1**	49.5:49.5:1 DOTAP/DOPE/ DSPE-PEG + NAMs	121 ± 9	0.20 ± 0.02	33 ± 3	41 ± 8	51 ± 11
**Rh-LS 1 mM**	47:47:1:5 DOTAP/DOPE/ DSPE-PEG/Rh-PE	111 ± 7	0.200 ± 0.002	36 ± 2	n/a[c]	n/a[c]
**Rh-LS 5 mM**	49:49:1:1 DOTAP/DOPE/ DSPE-PEG/Rh-PE	117 ± 6	0.19 ± 0.01	40 ± 1	n/a[c]	n/a[c]
**Rh-LS 10 mM**	49.25:49.25:1:0.5 DOTAP/DOPE/ DSPE-PEG/Rh-PE	107 ± 5	0.17 ± 0.01	36 ± 1	n/a[c]	n/a[c]

[a]%LE (E1)- loading efficiency calculated using Equation 1 of the methods section.

[b]%LE (E2)- loading efficiency calculated using Equation 2 of the methods section.

[c]n/a- not applicable, unloaded liposomes did not contain NAMs in the formulation.

Interaction between rhodamine-labeled liposomes (Rh-LS) and bacteria

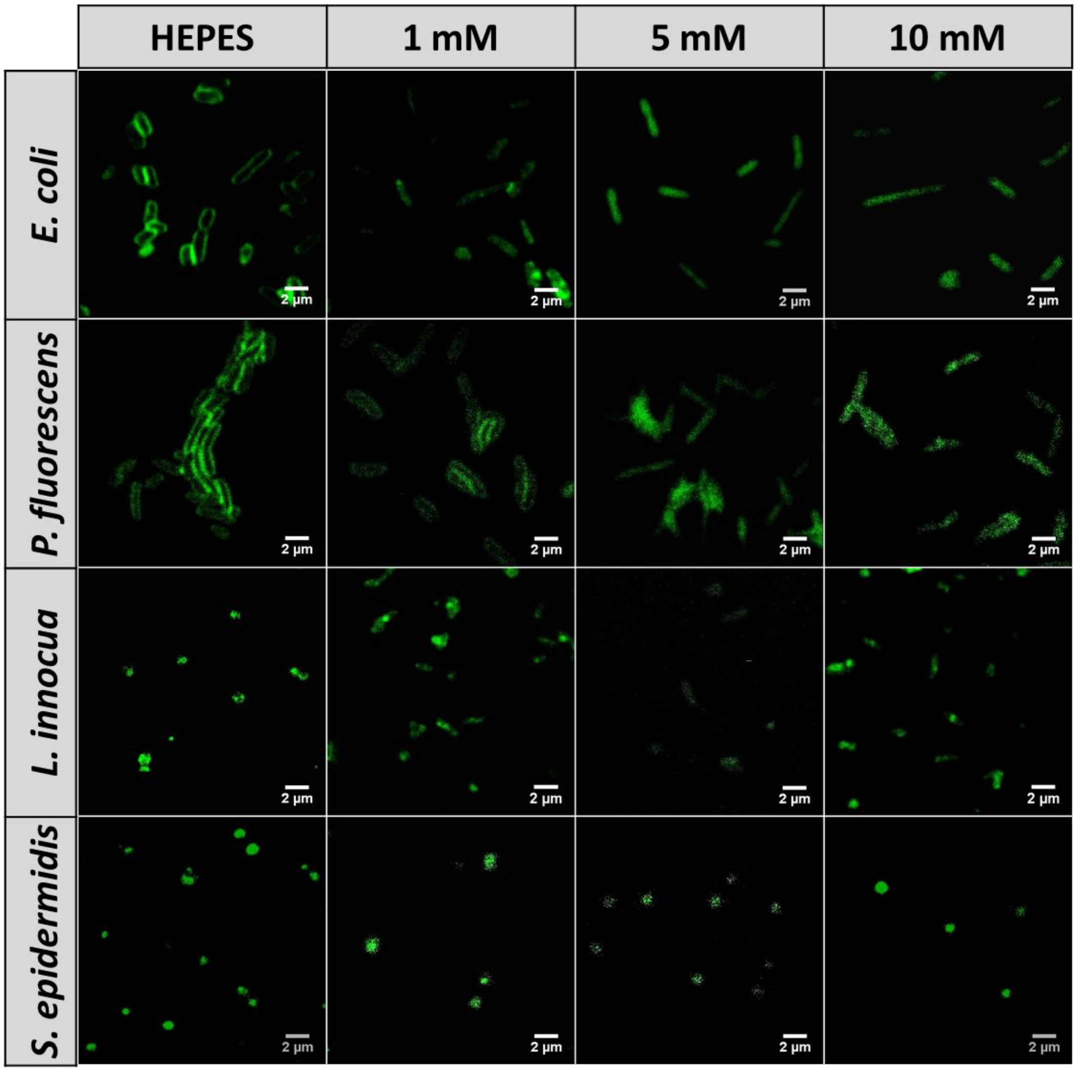

Figure S4. SynaptoRed C2 localization in *E. coli*, *P. fluorescens*, *L. innocua* and *S. epidermidis* upon fusion of Rh-LS at 1, 5 and 10 mM with bacterial cell envelopes and imaged by CLSM.

Bacterial surface zeta-potential

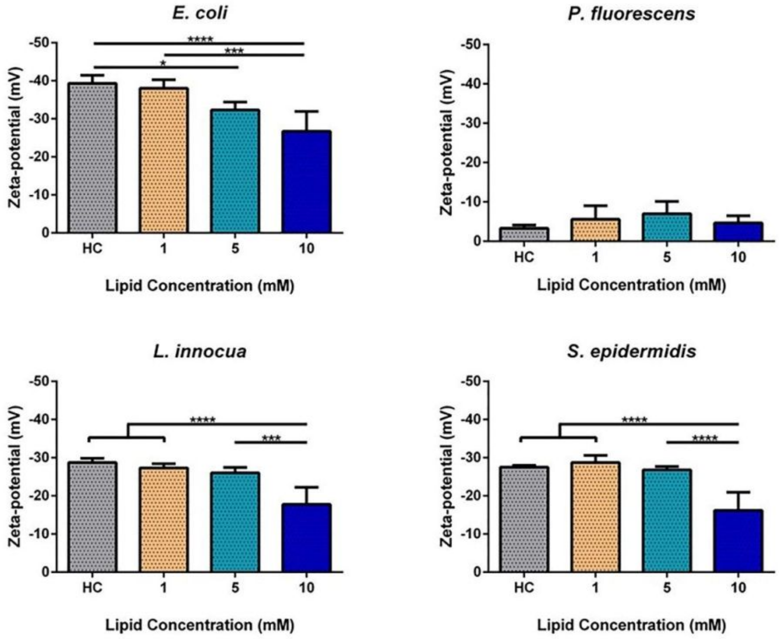

Figure S5. Bacterial surface zeta-potential of *E. coli*, *P. fluorescens*, *L. innocua* and *S. epidermidis* exposed to HEPES (HC, HEPES control) and 1, 5 and 10 mM of unloaded liposomes (LS). The results were plotted as mean ± standard deviation of five repeated assays. Brackets and lines indicate the groups being compared.
